# Pyrocarbon arthroplasty in acute unreconstructable radial head fractures: mid-term to long term results

**DOI:** 10.1186/s10195-018-0499-6

**Published:** 2018-08-23

**Authors:** F. Javier Ricón, Francisco Lajara, Alfonso Fuentes, María Luz Aguilar, Alberto Boix, Juan A. Lozano

**Affiliations:** 1 Orthopaedic and Trauma Surgery Department, Hospital Vega Baja de Orihuela, Calle País Valenciano, 14–B21, 03300 Orihuela, Alicante Spain; 2 Orthopaedic and Trauma Surgery Department, Hospital Vega Baja de Orihuela, Plaza de la Purisima, 12, 30640 Abanilla, Murcia Spain; 3 Orthopaedic and Trauma Surgery Department, Hospital Vega Baja de Orihuela, C/Conde de Floridablanca 48, Urbanización Altorreal, 30506 Molina de Segura, Murcia Spain; 4 Orthopaedic and Trauma Surgery Department, Hospital Vega Baja de Orihuela, C/Conquistador Pizarro, 6, 03360 Callosa de Segura, Alicante Spain; 5 Orthopaedic and Trauma Surgery Department, Hospital Vega Baja de Orihuela, C/Marqués de Molins 45, 03130 Santa Pola, Alicante Spain; 6 Orthopaedic and Trauma Surgery Department, Hospital Vega Baja de Orihuela, C/Limón 26, 03300 Orihuela, Alicante Spain

**Keywords:** Elbow, Radial head, Fracture, Arthroplasty, Results, Pyrocarbon

## Abstract

**Background:**

The aim of this study is to describe the mid-term radiological findings appearing in patients with a pyrocarbon radial head prosthesis, and to correlate them to patient symptoms.

**Materials and methods:**

We review 18 patients who underwent radial head implantation of the MoPyC prosthesis between 2004 and 2015, due to unreconstructible radial head fractures. The clinical outcomes were assessed with Mayo Elbow Performance Score (MEPS). Range of motion, pain, and elbow radiological assessments were recorded. A non-parametric, statistical analysis was carried out to assess the radiological findings with the clinical outcomes.

**Results:**

We have found that after a mean follow-up of 6.5 years (2–11 years), patients have recovered a median flexion arch of 113°, therefore 77% are classed as satisfactory outcomes and the average MEPS score is 89.5. The presence of periprosthetic changes on X-ray is highly frequent—we found radiolucent lines in 38% of cases, radial neck re-absorption in 83%, and arthrosic changes in 78%. However, the differences found when correlating these changes with the clinical results have not been statistically significant (*p* > 0.05).

**Conclusions:**

Satisfactory outcomes can be expected midterm when using pyrocarbon prostheses in around 75% of the cases. We consider radial neck re-absorption to be a sign of good stem osteointegration, whereas progressive radiolucencies and loss of the ballooning of the stem legs are signs of bad prognosis in our series.

**Level of Evidence:**

IV retrospective case series.

## Introduction

The use of radial head prostheses for unreconstructible radial head fractures is becoming increasingly widespread due to the satisfactory results reported in the literature, which occur in the majority of the series in more than 70% of the cases. This is also due to the poor results obtained with isolated resection of the radial head without replacing it, which may result in longitudinal radial instability, chronic elbow instability, increases valgus angle, etc. [1, 2]. To avoid these poor results we must replace the radial head with a prosthesis, being especially indicated if accompanied by injuries of the medial collateral ligament, or injuries of the lateral collateral ligament with associated coronoid process fractures or lesions of interosseous membrane and radioulnar distal joint [3, 4].

There are different types of prostheses in the market depending on their composition—silastic, vitallium, metallic, pyrocarbon, etc.; also depending on their design—modular or monoblock, unipolar or bipolar, cemented or press-fit, etc. [5]. Within modular and unipolar implants, we focus on the radial head pyrocarbon prosthesis Mopyc (Bioprofile-Tornier, Cedex, France), which is fixated with a press-fit mechanism. The difference with the other radial head prostheses, apart from the pyrocarbon composition of the radial head, is that the prosthesis stem has an inner screw that, after implantation, creates an expansion against the cortical area of the proximal radius as the screw is tightened.

The functional outcomes achieved with this prosthesis are good or excellent in between 77 and 97% of cases [6–10]. As their use and follow-up time increases, the number of complications and radiological findings also increase; although some of them, such as re-absorption of the radial neck [11] do not seem to bear any clinical relevance, we are not aware of the repercussions of the others on the patients’ clinical outcome.

Therefore, the aim of this study is to describe the mid-term radiological changes taking place in the elbow after implantation of a radial head pyrocarbon prosthesis, and to correlate them to the patients’ clinical situation.

## Materials and methods

We introduce a retrospective cohort of patients with a Mopyc-type radial head prosthesis acutely implanted in our service between April 2004 and February 2015, following an unreconstructible radial head fracture. We have excluded those with a follow-up shorter than 24 months, those presenting a previous injury of the elbow where the prosthesis was implanted, and those where radial head replacement was not undertaken primarily. Eighteen patients attended the clinical and radiological assessment.

The patients’ average age was 48 years (31–71 years), 13 were male and 5 were female. The radial head fracture was considered to be type III in all cases, according to Mason’s classification [12]. Table 1 details all the associated injuries on the elbow and the treatment for each one of them.

**Table 1:** Data on the patients and associated elbow injuries and their treatment

Case	Sex	Age (years)	Mechanism	Dislocation	Coronoid fracture	Treatment	Proximal ulna fracture	Treatment	Ligament repair
1	M	50	Precipitated	No			Simple	2KW + cerclaje	
2	M	35	Sport	Yes	Tip	Bone anchor			Bone anchor LLC
3	M	45	Simple fall	No					Bone anchor LLC
4	M	37	Simple fall	No					Bone anchor MCL
5	F	67	Simple fall	No	Tip	None	Simple	2KW + cerclaje	
6	M	41	Sport	No					
7	F	58	Simple fall	Yes					Bone anchor LLC y MCL
8	M	63	Simple fall	Yes	Tip	Screw			Bone anchor LLC
9	M	47	Simple fall	No	Tip	Screw			
10	F	42	Simple fall	No					
11	F	31	Simple fall	No	Base	Plate	Complex	Plate	Bone anchor LLC + Ex -fix
12	F	64	Simple fall	No					Bone anchor LLC and MCL
13	M	59	Simple fall	No			Simple	2KW + cerclaje	
14	M	71	Simple fall	Yes	Tip	Screw			Bone anchor LLC and MCL
15	M	30	Sport	Yes	Tip	Screw			Bone anchor LLC
16	M	39	Sport	No	Tip	Plate	Dyaphisis	Plate	Bone anchor LLC
17	M	44	Simple fall	No					
18	M	42	Sport	No	Base	Plate	Complex	Plate	

*M* male, *F* female, *KW* Kirschnner wires, *LLC* lateral ligamentous complex, *MCL* medial collateral ligament, *Ex-fix* external fixator

During surgery, we used lateral access via the interval described by Kocher or Kaplan [13, 14] to fit the radial head prosthesis in all patients. The prosthesis used in every case was a Mopyc, which is a modular unipolar prosthesis with a pyrocarbon head and a press-fit non-cemented stem [6].

The clinical outcomes were assessed by an independent observer (AB), who used the VAS (Visual Analogue Scale) to register the degree of pain, particularly on the radial side of the elbow, as well as range of motion, and completed the Mayo Elbow Performance Score (MEPS).

**Fig. 1:**
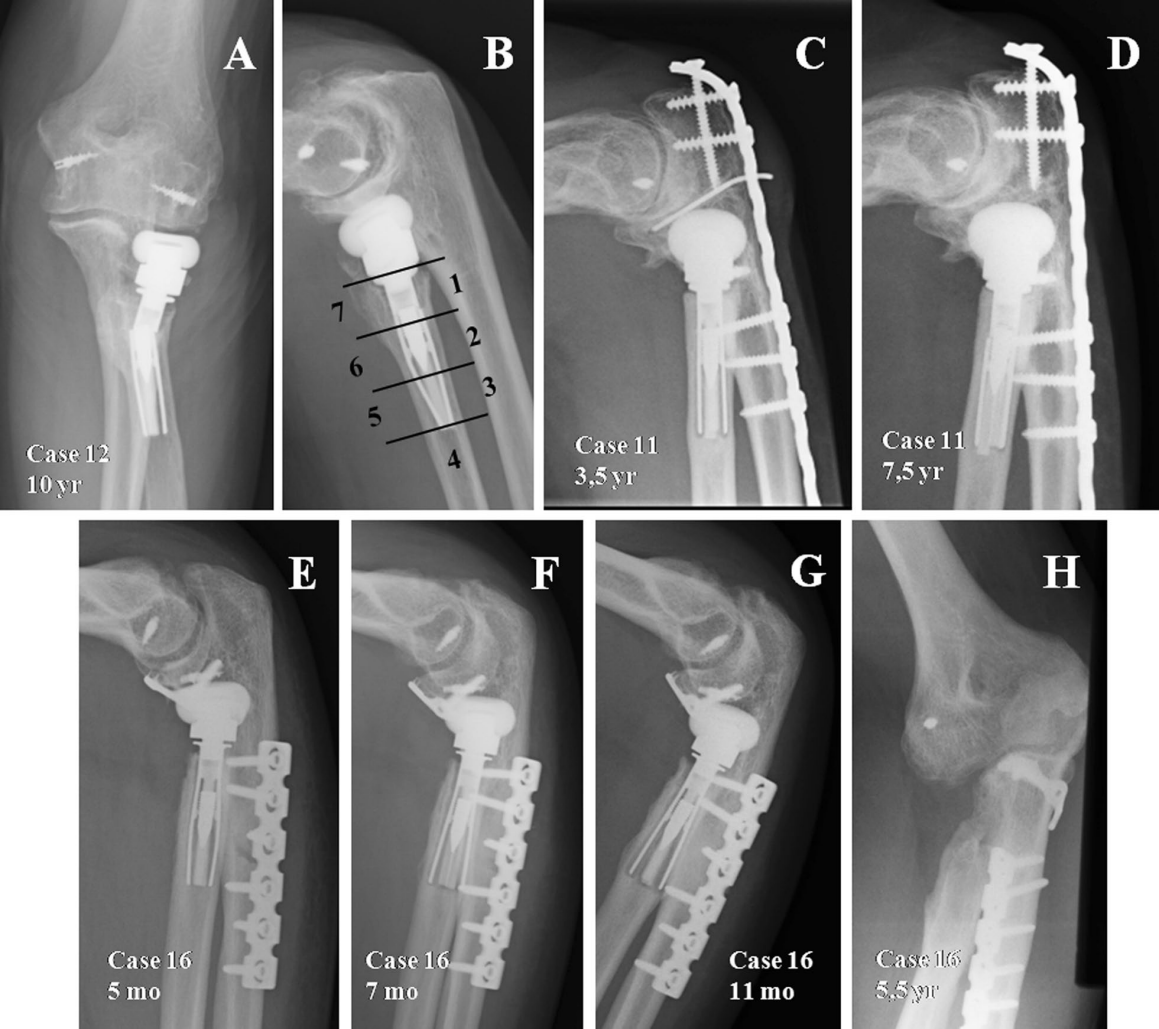
**A**, **B** Elbow movements have caused a concentration of stress in the transition point between the part that is well fixed and the part that is mobile, which has caused the stem to break in that point. **C**, **D** The non progressive cases do not register this stress-shielding phenomenon, therefore the stem has never been well osteointegrated due to a mild instability that has never stopped until finding balance (looser prosthesis). **E**–**H** Example of progressive radiolucent lines with stress-shielding phenomenon IIB, where the stem was completely loose, and pain improved after prosthesis removal; therefore we recommended prosthesis removal without replacement in case of symptomatic loosening
- The presence of radial neck re-absorption, following the classification described by Chanlalit et al. [11] (Table 2). For those cases with complete circumferential re-absorption, their magnitude was registered and classed as mild (< 2 mm), moderate (between 2 and 5 mm) and severe (> 5 mm). The time elapsed between surgery and the radiological appearance of re-absorption were also registered.

In order to assess radiological outcomes, an anteroposterior and lateral X-ray of the elbow that had been operated on was taken and analysed, along with those X-rays carried out throughout follow-up, by three independent assessors (AF, MLA, FL) who registered the following parameters:

- The presence of radiolucent lines around the stem, using the area-based method described by Grewal et al. [15] (Fig. 1B). The lines’ width at the largest point was also registered in order to classify them as mild (< 1 mm), moderate (between 1 and 2 mm) and severe (> 2 mm). The lines were considered to be progressive when radiolucency expanded to a different area or when its width increased by 1 mm.

**Table 2:** Chanlalit’s radial neck resorption classification

Stage	Description
I	Cortical bone thinning
IIA	Partially exposed stem
IIB	Circumferentially exposed stem
III	Mechanical or impending failure
- The existence of arthrosic changes, according to the classification by Broberg and Morrey et al. [16], thus registering the presence of an osteophyte on the medial ulnohumeral side and the onset of changes in the capitellum, such as osteolysis, erosion or flattening.

Lastly, the association between radiological findings and the clinical and functional outcomes has been analised. Non-parametric tests have been used due to the limited number of cases (*n* < 30 patients). The *U*-Mann–Whitney test has been used for the quantitative variables, indicated for comparing mean rates in two independent unrelated samples. For the qualitative/dichotomous variables, the comparison has been carried out with contingency tables, Pearson’s Chi square test, and Yates and Fisher’s corrections when appropriate.

## Results

After an average follow-up of 79.8 months (24–130 months), seven out of the 18 patients attending check-ups reported lateral elbow side pain. For three of them, the intensity of pain was considered mild, and moderate for the remaining four. Three of the patients with moderate pain required removal of the prosthesis, so the clinical and radiological assessment was carried out using data gathered before prosthesis removal. For one patient, the pain was deemed to be caused by prosthesis overstuffing, so she underwent surgery to remove the prosthesis 6 years after implantation. During surgery, only the radial head and the neck could be removed, as the stem was perfectly osteointegrated. The patient’s symptoms disappeared, and at the end of follow-up a 100-point MEPS score was achieved. In the other two cases there were symptomatic loosening of the prosthesis stem, so all the component parts of the prosthesis were removed. One of them had an 85-point MEPS score with mild pain in the radial area of the elbow at the end of follow-up, and the other one was a more complex case, with significant joint destruction caused by a disease of the particles.

The median flexion arch achieved was 113° (90°–130°). The maximum flexion achieved ranged between 110° and 130°, with an average of 127°, and the mean extension deficit was 15° (0°–40°). Pronation was recovered completely in all patients, except for two who suffered a 20° loss. Supination was worse affected—mean supination of 77° with a 50° to 90° range was achieved. One patient developed radio-ulnar proximal synostosis, requiring surgical cleaning of calcifications, subsequently improving prono-supination of the forearm with a 20° deficit on each end.

The average MEPS score was 89.5 points with a 55–100 point range. According to MEPS score, 11 outcomes were excellent (61%), 3 were good (16.5%), 3 regular (16.5%) and 1 poor (6%) (Table 3).

**Table 3:** Functional results

Case	Follow-up (months)	MEPS	Pain	Flexion	Extension lag	Pronation	Supination
1	130	100		130	− 15	90	90
2	106	100		130	0	90	90
3	54	100		130	0	90	70
4	41	85	Mild	130	0	90	75
5	122	70*	Moderate	130^t^	0^t^	90^t^	90^t^
6	55	100		130	0	90	90
7	115	100		120	− 15	90	80
8	73	100		130	− 20	90	70
9	38	75	Mild	125	− 15	70	50
10	99	100		120	− 10	90	70
11	92	85	Mild	130	− 40	90	80
12	120	100		130	− 15	90	90
13	115	100		110	− 20	90	90
14	26	55*	Moderate	100^t^	− 10^t^	80^t^	70^t^
15	83	100		130	− 30	90	60
16	69	70*	Moderate	130^t^	− 20^t^	90^t^	50^t^
17	53	100		130	− 15	90	85
18	24	70	Moderate	130	− 30	70	70

*MEPS* Mayo Elbow Performance Score

* MEPS before prosthesis removal, ^t^ range of motion at final follow up after prosthesis removal)

There were 7 cases with radiolucent lines around the stem. Three were considered partial, affecting at least two of the areas described by Grewal [10]. All of them were classified as mild and not progressive. The other 4 radiolucencies affected the whole surface area of the stem. They were mild and non-progressive in two cases, and severe and progressive in the remaining two. In these four patients with complete radiolucencies around the stem, we have observed on X-ray a loss of the ballooning of the stem at the level of the expandable screw, reflecting a flaw in the osteointegration of the stem (Fig. 1).

**Fig. 2:**
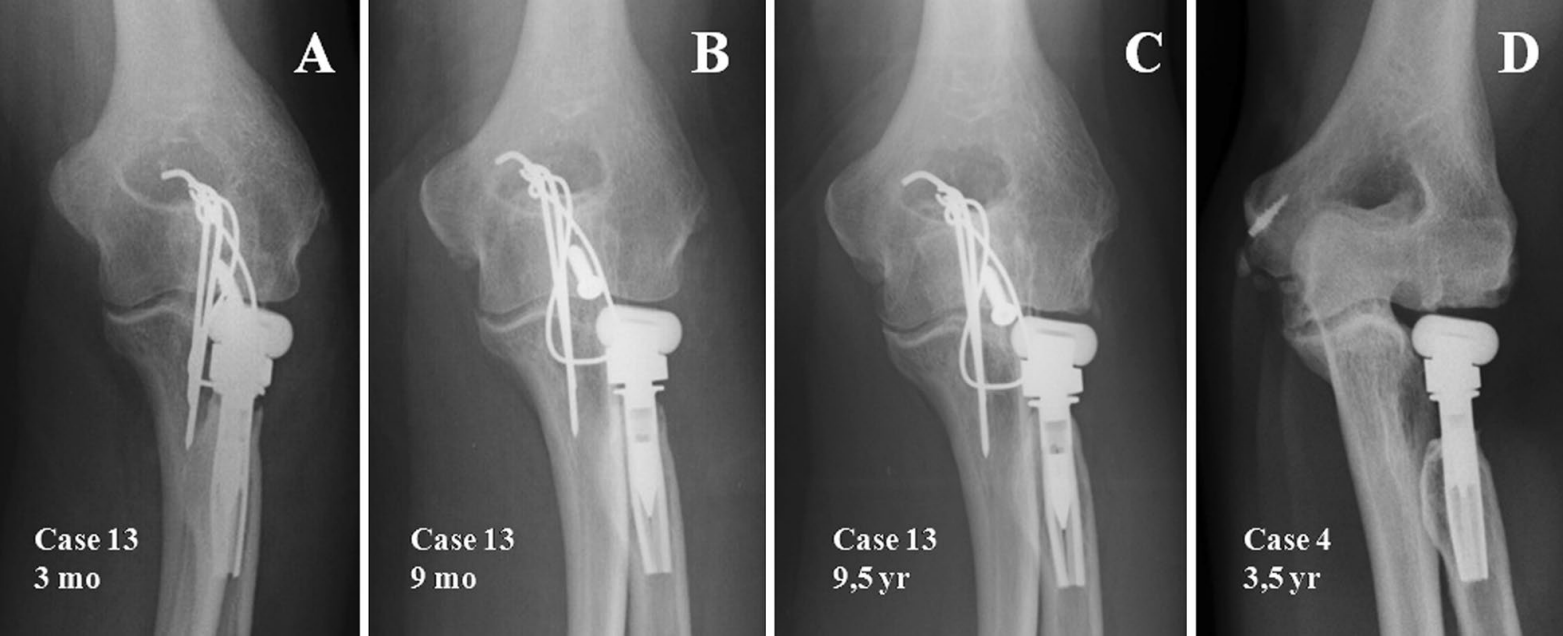
**A**–**C** Initially, there is type IIB reabsorption of the radial neck, which stabilises after 9 months with no evident progression until the end of the follow up. **D** Type II severe circumferential reabsorption, i e larger than 5 mm

In 5 cases there was no re-absorption on the radial neck. In the remaining 72% there was neck re-absorption—one case had thinning of the cortical area of the radial neck (stage 1); another case saw a partial re-absorption of the radial neck, i.e. a stage IIA; the 11 remaining cases had a complete re-absorption of the radial neck, i.e. stage 2B; of these, it was deemed to be mild in 5 cases, moderate in 5 other cases and severe in one. Their onset was early, and they were visible in every case during the first year after prosthesis implantation (Fig. 2).

**Fig. 3:**
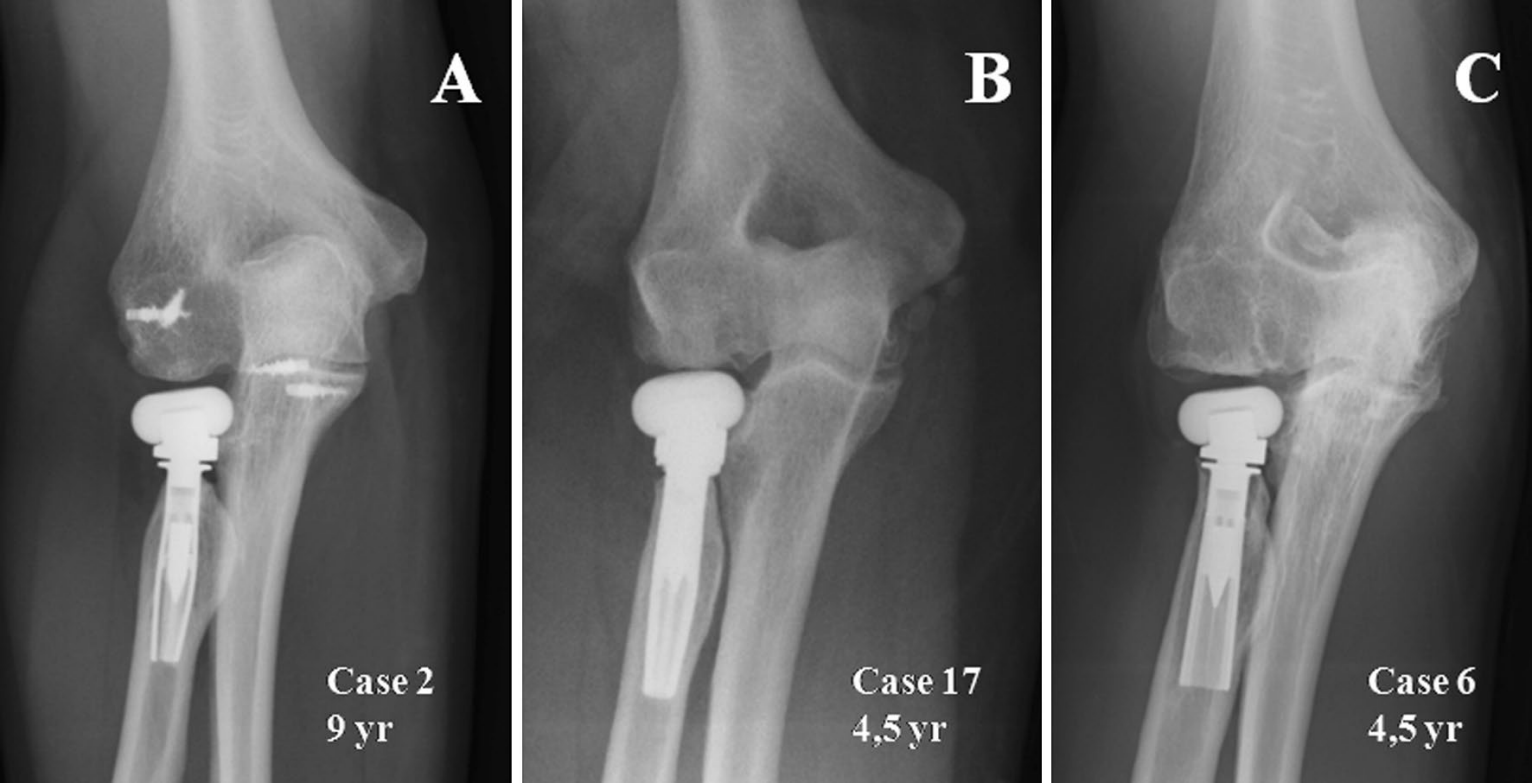
**A** Case 2, not showing degenerative changes after 106 months, nor changes in the capitellum or medial ulnohumeral osteophyte. **B** X-ray after 53 months in another patient with type I degenerative changes, according to Brian and Morrey’s classification, with slight changes in the capitellum and no medial osteophyte. **C** In this case there are severe changes in the capitellum, as well as osteophyte in the medial area of the ulnohumeral joint. We can also see a loss of press fit of the stem, as well as non-progressive radiolucent lines

During assessment of degenerative changes, we found that there were none in 3 cases, in 5 cases they were considered mild, in 9 cases they were moderate, and severe in one case, which was the patient who developed joint destruction caused by disease of the particles (Fig. 3). If we evaluate the capitellum individually, we find that at the end of follow-up, in 2 cases the capitellum was not affected, whereas in the remaining 16 cases it was subjected to changes (Fig. 3). When we assess the medial side of the joint, looking for an ulnohumeral osteophyte, we observe that this is present in 10 cases.

There are two cases of broken prosthesis stem: one patient lost the press-fit, and one of the stem legs was broken. In the other one, there was a complete break above the expanding screw. The radiological assessment is registered in Table 4.

**Table 4:** Radiological results

Case	Radial neck resorption		Radiolucent lines		Press-fit loss	Stem breakage	Degenerative changes		
	Classification	Grade	Zone	Grade			Classification	Medial spur	Capitellum changes
1	1	Mild			No		2	Yes	Yes
2	2B	Mild			No		0	No	None
3	2B	Mild	2, 6	Mild	No		0	No	None
4	2B	Severe			No		1	Yes	Yes
5	2B	Moderate			No		2	Yes	Yes
6	0	None	1, 2, 3, 4, 5, 6, 7	Mild	Yes		2	Yes	Yes
7	0	None			No		1	Yes	Yes
8	2B	Moderate			No		2	Yes	Yes
9	0	None			No		1	Yes	Yes
10	2B	Moderate			No		0	No	Yes
11	0	None	1, 2, 3, 4, 5, 6, 7	Mild	Yes	Stem leg	2	No	Yes
12	2A	Mild	1, 7	Mild	No	Proximal stem	2	No	Yes
13	2B	Mild	2, 6	Mild	No		1	Yes	Yes
14	2B	Moderate	1, 2, 3, 4, 5, 6, 7	Severe	Yes		3	No	Yes
15	0	None			No		2	Yes	Yes
16	2B	Mild	1, 2, 3, 4, 5, 6, 7	Severe	Yes		2	No	Yes
17	2B	Mild			No		1	No	Yes
18	2B	Moderate			No		2	Yes	yes

When completing the statistical analysis, trying to find a correlation between the clinical and the radiological situation, we found that moderate pain was present in all patients with progressive radiolucency; however, 31% of patients who did not have radiolucency, did report pain. Out of the 13 patients with radial neck re-absorption, 38% reported pain, while the remaining 62% did not have symptoms. We do not see a link between radial neck re-absorption, changes in the capitellum, or a medial ulnohumeral osteophyte, with poorer clinical results as measured by MEPS score, or with a poorer range of motion, although there is a non-significant trend towards extension deficit in cases with changes in the capitellum (*p* = 0.054).

## Discussion

The prosthesis used in this study was a MOPYC, which is a modular prosthesis with three component parts—a pyrocarbon head in three different sizes, a titanium neck with a 15° angulation, and a stem, also titanium, non cemented and expandable, both with 4 available sizes [6]. The outcomes achieved in the study are comparable to those found in other literature referred to this prosthesis and concerning other prosthesis designs. For example, Delcloux’s [17] review analysing over 30 series where radial heads prostheses have been implanted, finds a mean rate of successful outcomes of 81% for early implantation, which ranges between 61 and 100% [6–11, 15, 18–31]. In our project, we have a 77% rate of good or excellent results after an average follow-up of 6 years.

As proposed by O’Driscoll [32], pain in the proximal area of the forearm during follow-up, when it is located on the radial side and gets worse when carrying weight or exercising strength, is a symptom that should make us suspect a loosening of the prosthesis stem or another type of complication, particularly when the pain is moderate in intensity. In 3 out of our 4 cases where pain intensity was moderate, complications developed requiring total or partial removal of the radial head prosthesis. On the other case, he is awaiting prosthesis removal.

We believe, like Chanlalit [11] and Gauci [19], that radial neck re-absorption is caused by a stress-shielding mechanism. This extensively-documented phenomenon concerning well-implanted femoral stems is caused by a change in load transmission, in such a way that this is transmitted through the implant and not through the bone, thus causing bone loss around the implant. Popovic [20] raises the possibility that this bone loss is secondary to polyethylene wear, but the prosthesis we use does not contain polyethylene, so we have discarded this theory. Chanlalit [11] finds this process in 65% of his prostheses—this rate is very close to our own 72%. When we link this to the onset of pain or to functional outcomes, we find no statistically significant differences. Therefore it seems that there is no relationship between radial neck re-absorption and poor outcomes, as pain. It is registered in every case before the end of the first year of follow-up, meaning it is an early sign of good prosthesis fixation (Fig. 2A–C). The great problem with this phenomenon is whether it can jeopardise the right fixation of the stem, and predispose to the appearance of loosening or periprosthetic fractures [33], but we haven’t found this association in our series, even on the patient where re-absorption was considered of severe intensity, i.e. larger than 5 mm (Fig. 2D).

Upon revision of the rate of appearance of radiolucent lines, we see that it is highly variable depending on the type of prosthesis. When the prosthesis employed is smooth-stemmed implanted with a loose press-fit [15, 25–28], the presence of radiolucent lines is registered in between 100 and 50% of cases. As regards bipolar cemented prostheses, Popovic [20] finds radiolucencies in the 53% of his patients after a mean follow-up of 8.4 years. Lastly, with concern to press-fit implanted prostheses like ours, Flinkkila [28] finds radiolucency lines in 32% of his cases after a mean follow-up of 53 months, whereas Shore [29] finds radiolucency lines in 54% of his cases after 8 years’ mean follow-up. The average radiolucencies documented in our series is 38%—really high compared to the rest of articles employing this same prosthesis (around 6%) [6–9], which could be explained by the shorter follow-up of these other series. In his article, Popovic [20] makes a distinction between early radiolucencies due to a flaw in the cementation technique (20%) and progressive ones, due to mechanical factors, that give way to progressive osteolysis with the ultimate loosening of the prosthesis, which happen in 29% of his cases. If we make a careful evaluation of radiolucencies in our cases, we can also divide them in several types: in 3 cases (16%) there are partial radiolucency lines affecting at least two areas of the stem, in two cases (11%) they have complete radiolucency lines where patients are relatively asymptomatic and have good elbow functionality, and in the other two cases (11%), radiolucent lines around the whole of the stem are progressive and severe in intensity, with a moderate intensity pain (Table 5).

**Table 5:** Radiolucent lines description

Radiolucent lines	Stem changes	Progression	Pain	MEPS	Radial neck resorption	Elbow instability	Cases (no)
Partial	No	No	No	Excellent	2B	No	2
	Stem breakage	No	No	Excellent	2A	Mild	1
Complete	Ballooning loss	No	Mild	Excellent/good	0	Mild	2
	Ballooning loss	Yes	Moderate	Regular/poor	2B	Moderate/severe	2

If we correlate stress shielding to the presence of radiolucent lines, we find that in the two cases with partial radiolucencies in the middle of the stem, in areas 2 and 6 described by Grewal, they include 2B stress shielding with low intensity, and rate 100 points in the MEPS score, with no pain after 115 and 54 months’ follow-up. Their presence could be due to changes in load distribution, although it could also be attributed to non-symptomatic or progressive loosening of the initial stage. The other case presents a stem fracture, registering radiolucent lines in areas 1 and 7, just above the expandable screw. This translates as a lack of press-fit in the proximal area, the distal area being well fixated (Fig. 1A, B). Elbow movements during daily activities have caused a concentration of stress in the transition point between the part that is well fixed and the part that is mobile, which has caused the stem to break in that point. Radiologically, it appears alongside partial neck re-absorption, although it does not reach complete stress shielding, as it is the only case where we find stage IIA.

Complete radiolucencies can be progressive and symptomatic, and it could reflect gross or moderate elbow instability that gives way to the prosthesis loosening up or that can be asymptomatic and non-progressive radiolucencies, in which there could be a degree of elbow instability, albeit mild. This may also cause loosening of the stem with loss of press-fit, but this minor instability comes at a time when is compensated by the prosthesis movement inside the radius (looser-type prostheses), and finds a balance that prevents it from progressing like the previous type. The cases with progressive radiolucencies appear with circumferential radial neck re-absorption, thus suggesting a good stem osteointegration; however, if moderate or severe instability persists in that elbow, it causes a symptomatic loosening of the stem. In case 14, we have been able to confirm this approach, as dynamic radioscopy carried out prior to surgery to remove the prosthesis showed a posterior dislocation of both the ulna and the radial head when extending the elbow We have no radioscopy for the other case, only suppositions. During the surgery undertaken to remove the prosthesis, we saw in both cases that the stem was completely loose, and pain improved after prosthesis removal; one of the cases had a MEPS score without prosthesis of 85 points and mild pain, linked to effort-making, on the lateral side of the elbow; therefore we recommended prosthesis removal in case of symptomatic loosening (Fig. 1E–H).

The other two cases, non progressive, do not register this stress-shielding phenomenon, therefore the stem has never been well osteointegrated due to a mild movement that has never stopped until finding balance (looser prosthesis), as described by other authors [11, 19–22] (Fig. 1C, D). In all cases with complete radiolucency, there has been a loss of the ballooning of the stem, which we can consider as a radiological sign that forces us to think that the stem has lost its bone anchor.

Another radiological finding frequent in patients with radial head prostheses is that of changes in the capitellum. First described by Van Riet [34] in 2004, its presence can be attributed to initial trauma or factors related to the actual prosthesis, such as different elasticity module as regards the bone. There has been an attempt to improve the transmission of load bearing to the radial head by using pyrocarbon, because its elasticity module is lower than that of metallic prostheses and more similar to the bone [6]. However, in our series there are capitellum changes in 88% of cases, which is too high a figure to put into question whether the elasticity module of this prosthesis, with its special composition, is similar to that of the native bone (Fig. 3). If we link these capitellum changes to pain or functional outcomes, we do not find a statistically significant relationship, although there appear to be signs of a relationship between the extension deficit and changes in the capitellum. Therefore, although changes in the capitellum are not directly related to the onset of clinical symptoms, there are some cases where hyperpressure over the capitellum can cause moderate pain, as in case 5, where the prosthesis head was removed, thus improving the patient’s symptoms.

Our series presents the mid- to long term outcomes of a retrospective cohort of patients with Mopyc prostheses, although with fewer patients than that of Gauci et al. [19]. Nevertheless, in our essay we are analysing radiological changes and their relationship with pain, and MEPS score rates. Moreover, one of the other fortes of this project, despite the fact that it deals with a series of cases, is that these are consecutive and they were surgically intervened by members of the same service in a relatively short period of time. These features minimise the changeability in surgical technique and approach, as well as the learning curve. Due to the limited number of cases, the statistical analysis does not confirm to a statistically significant degree the relationship between the findings described. Nevertheless, the fact that this confirmation does not arise does not detract from the outcomes described: both quantitatively (mobility and MEPS score) and qualitatively (radiological changes). Another limitation of this study is that due to its retrospective nature, only the cases that attended check-ups were evaluated, which could mean a bias by overestimation or underestimation of the prostheses outcomes.

Satisfactory outcomes can be expected mid term when using Mopyc prosthesis in around 75% of cases. The appearance of periprosthetic radiological changes is frequent and has no clinical repercussions in most cases. We consider radial neck re-absorption to be a sign of good stem osteointegration, due to a stress-shielding mechanism. However, progressive radiolucency affecting the whole stem surface-area, and loss of the ballooning of the stem legs, are signs of a bad prognosis of the implant in our series.

## References

[1] Ikeda M, Oka Y (2000). Function after early radial head resection for fracture: a retrospective evaluation of 15 patients followed for 3–18 years. Acta Orthop Scand.

[2] Lindenhovius AL, Felsch Q, Doornberg JN, Ring D, Kloen P (2007). Open reduction and internal fixation compared with excision for unstable displaced fractures of the radial head. J Hand Surg Am.

[3] Morrey BF, Tanaka S, An KN (1991). Valgus stability of the elbow. A definition of primary and secondary constraints. Clin Orthop Relat Res.

[4] Schneeberger AG, Sadowski MM, Jacob HA (2004). Coronoid process and radial head as posterolateral rotatory stabilizers of the elbow. J Bone Joint Surg Am.

[5] Giannicola G, Sacchetti FM, Antonietti G, Piccioli A, Postacchini R, Cinotti G (2014). Radial head, radiocapitellar and total elbow arthroplasties: a review of recent literature. Injury.

[6] Allieu Y, Winter M, Pequignot JP, de Mourgues Ph (2006). Radial head replacement with a pyrocarbon head prosthesis: preliminary results of a multicentric prospective study. Eur J Orthop Surg Traumatol.

[7] Lamas C, Castellanos J, Proubasta I, Dominguez E (2011). Comminuted radial head fractures treated with pyrocarbon prosthetic replacement. Hand.

[8] Ricon FJ, Sanchez P, Lajara F, Galan A, Lozano JA, Guerado E (2012). Result of pyrocarbon prosthesis after comminuted and unreconstructable radial head fractures. J Shoulder Elbow Surg.

[9] Sarris IK, Kyrkos MJ, Galanis NN, Papavasilliou KA, Sayegh FE, Kapetanos GA (2012). Radial head replacement with the MoPyC pyrocarbon prosthesis. J Shoulder Elbow Surg.

[10] Abdulla IN, Molony DC, Symes M, Cass B (2015). Radial head replacement with pyrocarbon prosthesis: early clinical results. ANZ J Surg.

[11] Chanlalit C, Shukla DR, Fitzsimmons JS, An KN, O’Driscoll SW (2012). Stress shielding around radial head prostheses. J Hand Surg.

[12] Mason ML (1954). Some observations on fractures of the head of the radius with a review of one hundred cases. Br J Surg.

[13] Barco-Laakso R, Forcada-Calvet P, Ballesteros-Betancourt JR, Llusa-Perez M, Antuña S, Stanley D, Trail I (2012). Surgical approaches to the elbow. Operative elbow surgery.

[14] Morrey BF, Morrey BF (2009). Surgical exposures of the elbow. The elbow and its disorders.

[15] Grewal R, Mac Dermid JC, Faber KJ, Drosdowech DS, King GJW (2006). Comminuted radial head fractures treated with a modular metallic radial head arthroplasty study of outcomes. J Bone Joint Surg Am.

[16] Broberg MA, Morrey BF (1987). Results of treatment of fracture-dislocations of the elbow. Clin Orthop Relat Res.

[17] Delcloux S, Lebon J, Faraud A, Toulemonde J, Bonnevialle N, Coulet B (2015). Complications of radial head prostheses. Int Orthop.

[18] Moro JK, Werier J, MacDermid JC, Patterson SD, King GJ (2001). Arthroplasty with a metal radial head for unreconstructible fractures of the radial head. J Bone Joint Surg (Am).

[19] Gauci MO, Winter M, Dumontier C, Bronsard N, Allieu Y (2016). Clinical and radiological outcomes of pyrocarbon radial head prosthesis: midterm results. J Shoulder Elbow Surg.

[20] Popovic N, Lemaire R, Georis P, Gillet P (2007). Mid term results with a bipolar radial head prosthesis: radiographic evidence of loosening at the bone–cement interface. J Bone Joint Surg Am.

[21] Ashwood N, Bain GI, Unni R (2004). Management of mason type-III radial head fractures with a titanium prosthesis, ligament repair, and early mobilization. J Bone Joint Surg (Am).

[22] Dotzis A, Cochu G, Mabit C, Charissoux JL, Arnaud JP (2006). Comminuted fractures of the radial head treated by the Judet floating radial head prosthesis. J Bone Joint Surg (Br).

[23] Doornberg JN, Parisien R, van Duijn PJ, Ring D (2007). Radial head arthroplasty with a modular metal spacer to treat acute traumatic elbow instability. J Bone Joint Surg (Am).

[24] Fehringer EV, Burns EM, Knierim A, Sun J, Apker KA, Berg RE (2009). Radiolucencies surrounding a smooth-stemmed radial head component may not correlate with forearm pain or poor elbow function. J Shoulder Elbow Surg.

[25] Harrington IJ, Sekyi-Otu A, Barrington TW, Evans DC, Tuli V (2001). The functional outcome with metallic radial head implants in the treatment of unstable elbow fractures: a long-term review. J Trauma.

[26] Schnetzke M, Aytac S, Deuss M, Studier-Fischer S, Swartman B, Muenzberg M (2014). Radial head prosthesis in complex elbow dislocations: effect of oversizing and comparision with ORIF. Int Orthop.

[27] Zunkiewicz MR, Clemente JS, Miller MC, Baratz ME, Wysocki RW, Cohen MS (2012). Radial head replacement with a bipolar system: a minimum 2 year follow-up. J Shoulder Elbow Surg.

[28] Flinkkilä T, Kaisto T, Sirniö K, Hyvönen P, Leppilahti J (2012). Short to mid-term results of metallic press-fit radial head arthroplasty in unestable injuries of the elbow. J Bone Joint Surg Br.

[29] Shore BJ, Mozzon JB, MacDermid JC, Faber KJ, King GJW (2008). Chronic posttraumatic elbow disorders treated with metallic radial head arthroplasty. J Bone Joint Surg Am.

[30] Allavena C, Delclaux S, Bonnevialle N, Rongières M, Bonnevialle P, Mansat P (2014). Are bipolar radial head prostheses adapted for the treatment of complex radial head fractures? About 22 prostheses followed-up an average of 50 months. Orthop Trauma Surg Res.

[31] Celli A, Modena F, Celli L (2010). The acute bipolar radial head replacement for isolated unreconstructable fractures of the radial head. Musculoskelet Surg.

[32] O’Driscoll SW, Herald JA (2012). Forearm pain associated with loose radial head prostheses. J Shoulder Elbow Surg.

[33] Shukla DR, Fitzsimmons JS, An KN, O’Driscoll SW (2012). Effect of stem length on prosthetic radial head micromotion. J Shoulder Elbow Surg.

[34] Van Riet RP, Van Glabbeek F, Verborgt O, Gielen J (2004). Capitellar erosion caused by a metal radial head prosthesis: a case report. J Bone Joint Surg Am.

